# Features of cognitive impairment in affective disorders

**DOI:** 10.1192/j.eurpsy.2025.1024

**Published:** 2025-08-26

**Authors:** E. Markova, I. Goldina, B. Goldin

**Affiliations:** 1 Neuroimmunology Lab, State Research Institute of Fundamental and Clinical Immunology; 2 Department of Psychiatry, Narcology, Psychotherapy and Clinical Psychology, Novosibirsk State Medical University, Novosibirsk, Russian Federation

## Abstract

**Introduction:**

Iimpaired cognitive functions are an urgent problem of modern neurology and psychiatry. Cognitive failure appears to be as etiologically heterogeneous syndrome, characterized by a decrease in memory indicators below the age norm, with unimpaired intellectual functioning and well-preserved activity. The wide prevalence of affective disorders and the leading role of the cognitive component in the process of personal self-development determine the undoubted relevance of studying the frequency and severity of cognitive impairment in affective disorders.

**Objectives:**

The purpose of this work was to study the cognitive impairment frequency and features in patients with generalized anxiety disorder (GAD) or panic disorder with agoraphobia (PDA).

**Methods:**

The study included 25 patients with an established diagnosis of GAD and 20 patients PDA. The control group consisted of 20 conditionally healthy volunteers. Cognitive function was assessed based on complaints, a study of visual-constructive skills, naming, memory, attention, praxis, speech, abstraction, orientation, using the Mini-mental State Examination scale and the Montreal Cognitive Function Rating Scale. The severity of anxiety disorders symptoms was determined during a clinical interview by a psychiatrist, as well as using the Hamilton Anxiety Scale (HDRS), Beck Anxiety Scale (BAI).

**Results:**

All patients in contrast to the healthy participants showed signs of cognitive impairment of varying severity. When studying cognitive function, the following was found:

1. patients with PDA were characterized by mild non-dementia cognitive impairment and showed signs of rumination - a tendency to think through the same stressful event over and over again. No memory or attention impairments were identified;
2. in patients with GAD moderate cognitive impairment was observed, the severity of which was obvious to both patients and their relatives in the form of impaired attention and memory, also in the absence of signs of dementia. The spectrum of cognitive impairment manifestations included impaired attention, executive functions and memory disorders. These patients were characterized by perfectionism, decreased confidence in their own memory, anxious doubts and double-checks.

**Conclusions:**

Our data indicate that in all patients with affective disorders impaired cognitive functions are detected. GAD is characterized by a greater severity of cognitive deficits compared to those in PDA.

**Disclosure of Interest:**

None Declared

